# The effect of tumour size on drug transport and uptake in 3-D tumour models reconstructed from magnetic resonance images

**DOI:** 10.1371/journal.pone.0172276

**Published:** 2017-02-17

**Authors:** Wenbo Zhan, Wladyslaw Gedroyc, Xiao Yun Xu

**Affiliations:** 1 Department of Chemical Engineering, Imperial College London, South Kensington Campus, London, United Kingdom; 2 Department of Radiology, Imperial College Healthcare NHS Trust, St Mary’s Hospital, London, United Kingdom; Academia Sinica, TAIWAN

## Abstract

Drug transport and its uptake by tumour cells are strongly dependent on tumour properties, which vary in different types of solid tumours. By simulating the key physical and biochemical processes, a numerical study has been carried out to investigate the transport of anti-cancer drugs in 3-D tumour models of different sizes. The therapeutic efficacy for each tumour is evaluated by using a pharmacodynamics model based on the predicted intracellular drug concentration. Simulation results demonstrate that interstitial fluid pressure and interstitial fluid loss vary non-linearly with tumour size. Transvascular drug exchange, driven by the concentration gradient of unbound drug between blood and interstitial fluid, is more efficient in small tumours, owing to the low spatial-mean interstitial fluid pressure and dense microvasculature. However, this has a detrimental effect on therapeutic efficacy over longer periods as a result of enhanced reverse diffusion of drug to the blood circulation after the cessation of drug infusion, causing more rapid loss of drug in small tumours.

## Introduction

A variety of therapeutic agents are routinely delivered by intravenous administration in clinical cancer treatments. The transport of therapeutic agents is determined by physicochemical properties of the drug and biologic properties of the tumour, including molecular structure of the drug, microvasculature density of the tumour and interstitial fluid pressure [1]. The biologic properties of a solid tumour, especially the density and distribution of tumour vasculature, could vary considerably depending on the particular tumour type, size and growth stage [2, 3]. Enlarged, tortuous and dilated microvessels are often found in tumours, leading to a variety of vascular network structures which may also evolve as tumours grow [4, 5]. It has been reported that large tumours have fewer microvessels than in small tumours [6].

Given the multiple processes involved in drug delivery and interactions between drugs and intratumoural environment, mathematical modelling has become an important tool to understand the limiting factors in effective delivery of anticancer drugs to solid tumours. A 1-D computational framework was developed by Baxter and Jain [7–9] to study the transport of fluid and macromolecules in solid tumours. A 2-D computational model was employed by Goh *et al* [10] to investigate the spatial and temporal variations of doxorubicin concentration in hepatoma. A similar study was carried out by Zhao *et al* [11] to address the effect of heterogeneous vasculature on interstitial transport in a 3-D embedded murine sarcoma model. The exchange of fluid between the circulatory system and tumour interstitium was studied by Soltanti *et al* [12] in idealized tumour geometries with various sizes and shapes, and the transport of F(ab’)_2_ from vasculature to extracellular space in these idealized models was examined in their subsequent work by assuming the same tissue properties for all tumours [13]. However, transcellular drug transport and cellular uptake were not included in these studies.

In the present study, the effect of tumour size on drug transport and its uptake by tumour cells are determined by means of 3-D computational modelling applied to realistic tumour geometries reconstructed from magnetic resonance images (MRI). The computational model incorporates the key physical and biochemical processes involved in drug transport from tumour vasculature to tumour interstitial space and across tumour cells. Tumours are treated as porous media and the vasculature density in each model is dependent on tumour size. Using the predicted intracellular drug concentration, anticancer efficacy is evaluated based on the percentage of viable tumour cells obtained by directly solving the pharmacodynamics equation corresponding to continuous infusion of doxorubicin.

## Mathematical models

In order to examine the interactions among multiple drug transport steps, tumour properties and drug properties, the current modelling platform consists of descriptions of interstitial fluid flow, convection and diffusion of drug in tumour interstitial space, transport of drug across cell membrane and a pharmacodynamics model. Tumour interstitium is modelled as a porous medium, with tumour vasculature being treated as a source term in the governing equations, without considering its geometric structure. The main assumptions are as follows: (1) the interstitial fluid is incompressible and Newtonian with a constant density and viscosity; (2) homogeneous transport properties in tumour; (3) uniform distribution of blood vessels and tumour cells in tumour tissue, with all cells being identical and stationary; (4) tumour growth is negligible within the simulation timeframe, so that all the physiological parameters and tumour geometry are independent of time.

**Fig 1:**
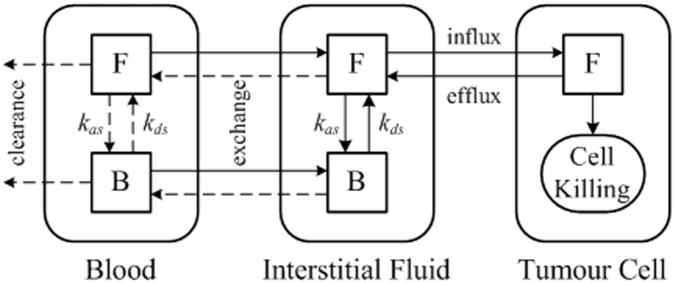
Schematic of drug transport model with continuous infusion of doxorubicin.

The mathematical models consist of the mass and momentum conservation equations for interstitial fluid flow, mass transfer equations for the free and bound drug, as well as equations describing the intracellular drug concentration and pharmacodynamics. Numerical solutions are obtained by solving the interstitial fluid flow equations first to provide the basic biomechanical environment for drug transport. This is followed by solution of the mass transfer equations for drug transport, which is described schematically in Fig 1 for direct infusion of doxorubicin, a commonly used anti-cancer drug. Briefly, the tumour region consists of three compartments: blood, extracellular space and tumour cells. Within each compartment, letters F and B represent free and bound doxorubicin, respectively.

The dynamics process in drug delivery includes drug association/disassociation with proteins at the rate *k*_*as*_ and *k*_*ds*_ respectively, drug exchange between blood and extracellular space, and influx/efflux of drugs from extracellular space to tumour cells. The rate of cell killing is governed by a pharmacodynamics model based on the predicted intracellular concentration of anticancer drugs. Detailed descriptions of the mathematical models have been reported elsewhere [14], a brief summary of the models is given in S1 Table.

### Model geometry

Five tumour models were reconstructed from images acquired from prostate cancer patients using a 3.0-Tesla MR scanner (DISCOVERY MR750, GE, Schenectady, New York, USA). Multislice anatomical images of the prostate were acquired in three orthogonal planes with echo-planer (EP) sequence, with each image comprising 256 by 256 pixels. Other imaging parameters are given in Table 1. All data were analysed anonymously and patient information was de-identified prior to analysis. The images were anonymized as they were saved from the P ACS system by the radiologist who acquired the images, and the archiving of the images for this study was done under anonymous patient numbers so that patients could not be identified away from the P ACS system. Formal ethical approval was not required for this retrospective study, as prior agreement was made to undertake computational modelling work using totally anonymised images without requiring further specific ethics committee agreement for individual patients. For this reason, written consent was not obtained from each individual patient to use their data in this specific study.

**Table 1:** MR imaging parameters.

	Pixel Size (mm)	Field of View (cm)	Slice Thickness (mm)	Repetition Time (ms)	Echo Time (ms)
Case 1	1.250	32.0	7.00	3675	85.7
Case 2	1.328	34.0	8.00	5150	69.6
Case 3	1.250	32.0	7.00	4000	84.4
Case 4	1.328	34.0	8.00	4825	69.6
Case 5	1.250	32.0	7.00	4000	91.5

**Fig 2:**
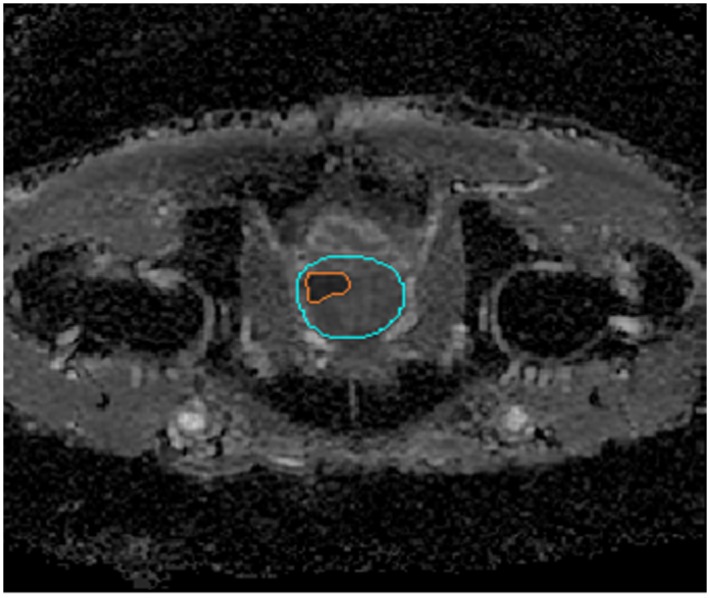
MR image of a tumour (in orange) and its surrounding tissue (in pale blue).

A sample MR image used for geometric reconstruction of the tumour models is shown in Fig 2 with the tumour region and its surrounding normal tissues. Transverse images are processed using image analysis software Mimics (Materialise HQ, Leuven, Belgium), and the tumours are segmented from its surrounding normal tissues based on signal intensity values. The resulting smoothed surfaces of the tumour and normal tissues are imported into ANSYS ICEM CFD to generate computational mesh for the entire 3-D volume.

**Fig 3:**
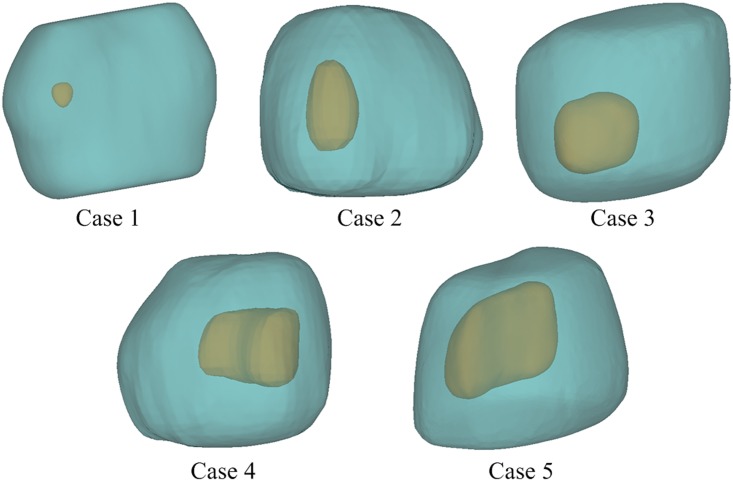
Reconstructed 3-D models showing tumours of varying sizes and their surrounding tissues.

The reconstructed models shown in Fig 3 are approximately 40~50 mm in length, but the tumour sizes are very different (see Table 2). The final computational mesh consists of 1,173,908, 1,446,643, 1,163,374, 1,216,811 and 1,474,027 tetrahedral elements for Case 1 to 5, respectively. These meshes have been obtained after mesh sensitivity tests to ensure grid independent solutions.

**Table 2:** Tumour size and the blood vessel surface area to tissue volume ratio (*S/V*) for each model.

Parameter	Unit	Case 1	Case 2	Case 3	Case 4	Case 5
Tumour size	mm^3^	45.46	909.08	2277.97	4162.89	7118.84
*S/V*	mm^-1^	25.28	13.80	11.46	10.15	9.10

### Model parameters

All the geometric and transport parameters are fixed within the simulation timeframe. Baseline values for model parameters are summarized in S2 and S3 Tables. Justifications for the choice of selected key parameters are given below.

#### Surface area of blood vessels per unit volume of tumour tissue (*S/V*)

**Fig 4:**
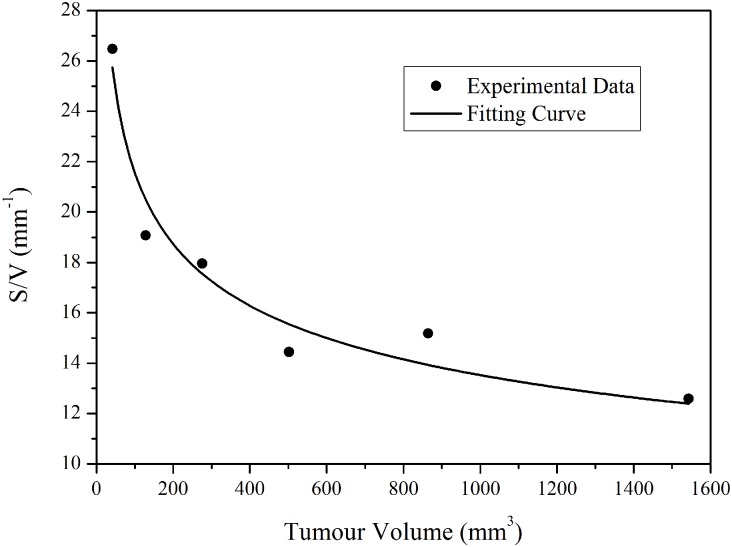
Estimation of blood vessel surface area to tissue volume ratio of tumours with various tumour sizes (experimental data extracted from [6]). For the best fitted curve using equation *y* = *ax*^*b*^ (a = 54.68, b = -0.2021).

This ratio reflects the microvasculature density. Pappenheimer *et al* measured *S/V* in normal tissues and found this to be 70 cm^-1^ [15], which was adopted by Baxter and Jain [7–9] in their computational studies. Hilmas and Gillette [6] measured *S/V* in breast tumours of different volumes and at different growth stages by using morphometric methods. Based on their data [6], the relationship between *S/V* and tumour size can be obtained as shown in Fig 4, from which the *S/V* value for each tumour model is derived according to its own size. Tumour volume and the corresponding *S/V* for each tumour model are summarized in Table 2.

#### Drug dose

Owing to the known toxicity of doxorubicin, the lifetime dose a patient can receive is approximately 550 mg per unit body surface area. For a patient of 70 kg body weight, a standard dose of 50 mg/m^2^ [16] is chosen for each treatment cycle.

#### Plasma pharmacokinetics (*C*_*v*_)

Doxorubicin concentration in blood plasma is modelled as an exponential decaying function of time. The form of equations depends on the infusion mode. For continuous infusion, a tri-exponential decay function is adopted based on the plasma pharmacokinetics of doxorubicin [17, 18].

$$\left\{ \begin{array}{l} C_{v}=\frac{D_{d}}{T_{d}}\left[\left(\frac{A_{1}}{\alpha_{1}}\left(1-e^{-\alpha_{1}t}\right)+\frac{A_{2}}{\alpha_{2}}\left(1-e^{-\alpha_{2}t}\right)+\frac{A_{3}}{\alpha_{3}}\left(1-e^{-\alpha_{3}t}\right)\right)\right]\;\left(t<T\right)\\ C_{v}=\frac{D_{d}}{T_{d}}\left[\frac{A_{1}}{\alpha_{1}}\left(e^{\alpha_{1}T_{d}}-1\right)e^{-\alpha_{1}t}+\frac{A_{2}}{\alpha_{2}}\left(e^{\alpha_{2}T_{d}}-1\right)e^{-\alpha_{2}t}+\frac{A_{3}}{\alpha_{3}}\left(e^{\alpha_{3}T_{d}}-1\right)e^{-\alpha_{3}t}\right]\;\left(t\geq T\right) \end{array}\right.$$ (1)

where *t* is time, *D*_*d*_ is the dose of total dose of administrated doxorubicin and *T*_*d*_ is the infusion duration. *A*_*1*_, *A*_*2*_ and *A*_*3*_ are compartment parameters and *α*_*1*_, *α*_*2*_, *α*_*3*_ are compartment clearance rates.

Free doxorubicin in plasma can easily bind with proteins, such as albumin. Greene *et al*. [19] found that approximately 75±2.7% doxorubicin is present in the bound form, and the percentage binding is independent of doxorubicin and albumin concentrations. Hence for direct infusion, free (*C*_*fp*_) and bound (*C*_*bp*_) doxorubicin concentrations in plasma are assumed to be 25% and 75%, respectively.

### Numerical methods

The mathematical models described above are coded in C programming language and implemented via user defined functions in ANSYS-Fluent, which is a finite volume based computational fluid dynamics (CFD) code (ANSYS Inc., Canonsburg, USA). The momentum and drug transport equations are discretised using the second order UPWIND scheme, and the SIMPLEC algorithm is employed for pressure-velocity coupling. The Gauss-Seidel smoothing method is used to update values at nodal points after each iteration step. Convergence is controlled by setting residual tolerances of the momentum equation and the drug transport equations to be 1×10^−5^ and 1×10^−8^, respectively.

In order to generate initial conditions for the transient simulation, the interstitial fluid flow equations are firstly solved to obtain a steady-state solution for the entire computational domain. The obtained pressure and velocity values are then used to initialise the simulation of drug transport and cellular uptake. The second order implicit backward Euler scheme is used for temporal discretisation, and a fixed time step size of 10 seconds is chosen. This time step is deemed sufficiently fine based on a time step sensitivity test. The initial doxorubicin concentrations are assumed to be zero in both tumour and the surrounding normal tissue.

There are two boundary surfaces in this model: an internal boundary between the tumour tissue and normal tissue, and the outer surface of the normal tissue. At the internal boundary, conditions of continuity in terms of interstitial pressure and fluid flux are applied. At the outer surface, a constant relative pressure of 0 Pa and zero flux of drug are assumed.

## Results

Numerical simulations have been carried out for 2-hour continuous infusion of 50 mg/m^2^ [16] doxorubicin, which corresponds to a standard treatment for a patient of 70 kg body weight [10]. The obtained interstitial fluid field is presented first as this provides the microenvironment for drug transport. This is followed by comparisons of doxorubicin concentration in the five tumour models to investigate the effect of tumour size on drug delivery. Finally, comparisons of predicted treatment outcomes are made based on remaining fractions of viable tumour cells.

### Interstitial fluid flow

**Fig 5:**
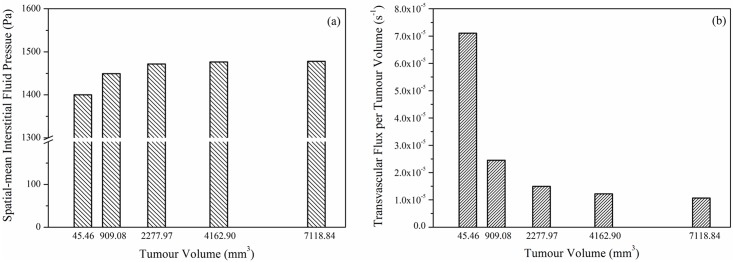
Interstitial fluid pressure distribution in tumour and normal tissue.

Because of abnormalities in tumour vasculature and the absence of lymphatic drainage, tumour interstitial fluid pressure (IFP) has been found to be higher than that in normal tissues [7–9, 20]. Contours of the predicted IFP at a representative cross-section for each of the five tumours are shown in Fig 5. IFP is uniformly high in the entire tumour except in a thin layer close to the tumour boundary. Owing to the low pressure in normal tissue, there is a large pressure gradient at the boundary between tumour and its surrounding normal tissue.

**Fig 6:**
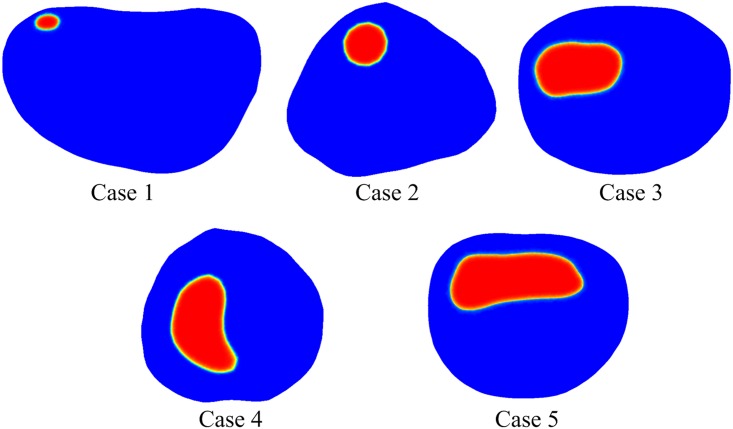
Spatial mean interstitial fluid pressure and transvacular flux per tumour volume as a function of tumour size.

The predicted spatial mean IFP and spatial mean transvascular flux per tumour volume in each tumour are shown in Fig 6(a) and 6(b), respectively. It can be observed that mean IFP varies with tumour size, with higher IFP in larger tumour. Since values for IFP in the main tumour body are the same for all five models (at 1534 Pa), the observed difference in mean IFP is caused by the different IFP gradient near the tumour boundary. It has been reported in a parameter sensitivity study that IFP gradient is steeper in larger tumour [7], which is consistent with the finding presented here.

The transvascular flux per tumour volume from blood to interstitium (*F*_*v*_) is calculated as the product of hydraulic conductivity of vessel wall, microvascular density as well as the difference between effective vascular pressure and IFP. Fig 6(b) shows that the spatial mean *F*_*v*_ is higher in small tumour. This is because: (1) the driving force for transvascular flow is high in small tumour owing to the low value of spatial mean IFP, and (2) higher *S/V* in small tumour further improves this exchange. However, referring to the spatial distribution of IFP in Fig 5, transvascular flow mainly occurs in a thin layer at the tumour/normal tissue interface owing to equilibrium being reached between IFP and the effective vascular pressure in tumour interior. Results also show that spatial mean IFP and transvascular flux per tumour volume are non-linearly related to tumour volume.

### Doxorubicin concentration

**Fig 7:**
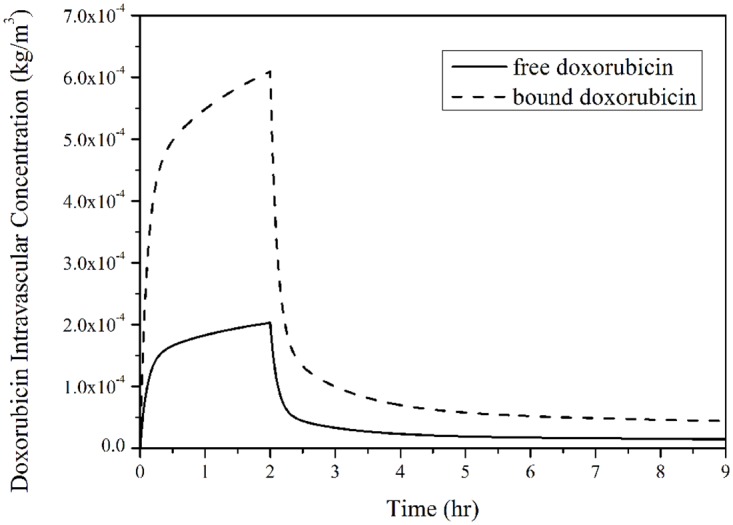
Free and bound doxorubicin concentrations in blood after administration as a function of time. (infusion duration = 2 hours, total dose = 50 mg/m^2^).

Intravascular concentrations of free and bound doxorubicin are shown in Fig 7. As expected, both free and bound Doxorubicin concentrations follow the exponential decay function described in Eq (1) and reach their peak values at the end of 2-hour infusion. This is followed by a rapid fall, after which there is a gradual and slow reduction as time proceeds. As prescribed, 25% doxorubicin is available in free form.

**Fig 8:**
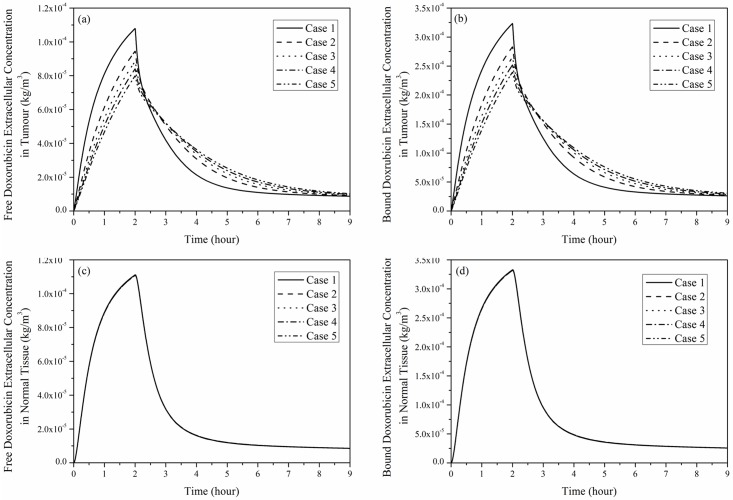
Spatial mean doxorubicin extracellular concentration as a function of time under 2-hour continuous infusion, dose = 50 mg/m^2^. (a) Free and (b) bound doxorubicin in tumour, (c) free and (d) bound doxorubicin in normal tissue.

Free and bound doxorubicin extracellular concentrations in tumour and its surrounding normal tissue are shown in Fig 8. Regardless of the tumour size, doxorubicin concentrations in tumour and normal tissue increase rapidly during the initial period after administration, reach their peaks and then fall to a low level after the end of infusion. The rate of change in doxorubicin concentration slows down with the increase in tumour size. This is because *S/V* is lower in larger tumour, leading to less drug exchange between blood and tumour interstitium. As shown in Fig 8(c) and 8(d), variation of tumour size has no obvious influence on drug concentration in normal tissue.

**Fig 9:**
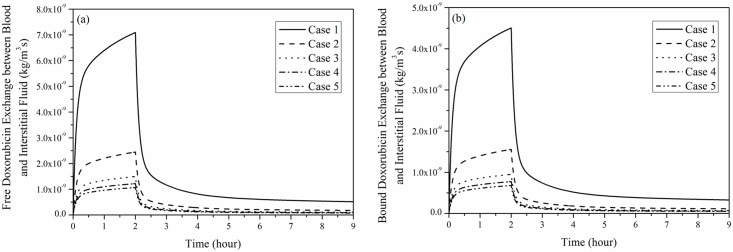
Transvascular exchange of doxorubicin by convection as a function of time. (a) free and (b) bound doxorubicin exchange (2-hour infusion, total dose = 50 mg/m^2^).

Fig 9 shows the transvascular exchange of drug by convection in each tumour, which follows the same pattern as shown in Fig 7, suggesting that intravascular concentration has a direct influence on transvascular transport of doxorubicin by convection. Results in Fig 9 also suggest that convective transvascular transport is more efficient in small tumour than in large tumour.

**Fig 10:**
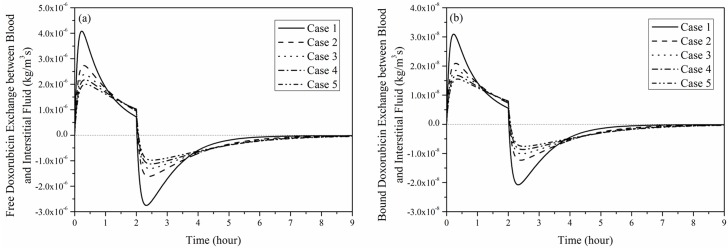
Transvascular exchange of doxorubicin by diffusion as a function of time in each tumour. (a) free and (b) bound doxorubicin exchange by diffusion (2-hour infusion, total dose = 50 mg/m^2^).

Regardless of the tumour size, transvascular transport of doxorubicin by diffusion shown in Fig 10 experiences the following stages: (1) rapid increase at the start of infusion, (2) gradual fall to a low but positive level at the end of infusion, (3) sharp fall to a negative level, and (4) slow recovery to zero.

Because the initial value of doxorubicin extracellular concentration is zero, the rapid increase in intravascular concentration results in a large concentration gradient between blood and the interstitial fluid when drug infusion starts, therefore, transvascular transport by diffusion increases rapidly to its peak. As the increase in blood concentration slows down, the transvascular concentration gradient is reduced, leading to a reduction in diffusive transport. However, transvascular diffusive flux remains positive until the end of drug administration, after which intravascular concentration drops rapidly as no more doxorubicin is supplied. As a consequence, transvascular concentration gradient falls to zero immediately. As doxorubicin is cleared from the intravascular space, its extracellular concentration becomes higher, causing transvascular exchange to reverse. Afterwards, there is a gradual fall in the negative concentration gradient as the extracellular concentration decreases, causing the diffusive transport to cease eventually.

Comparison of transvascular exchange of free and bound doxorubicin presented in Figs 9 and 10 indicates that transvascular flux of free doxorubicin is higher than that of bound drug. This can be attributed to the small osmotic reflection coefficient and high permeability of free doxorubicin, which ease the transvascular transport process. Results also show that the rate of change slows down as the tumour size increases, because the sparse microvasculature in large tumour reduces drug exchange between blood and interstitial fluid in tumour.

Comparison of convective and diffusive transvascular exchange presented in Figs 9 and 10 suggests that diffusion is the dominant transport mechanism by far (~3 orders of magnitude larger than convection). It is also worth noting that transvascular transport by convection mainly occurs in a thin layer at the interface between tumour and normal tissue. On the contrary, transvascular transport by diffusion takes place in the entire tumour and normal tissue owing to the concentration gradient across microvasculature walls.

**Fig 11:**
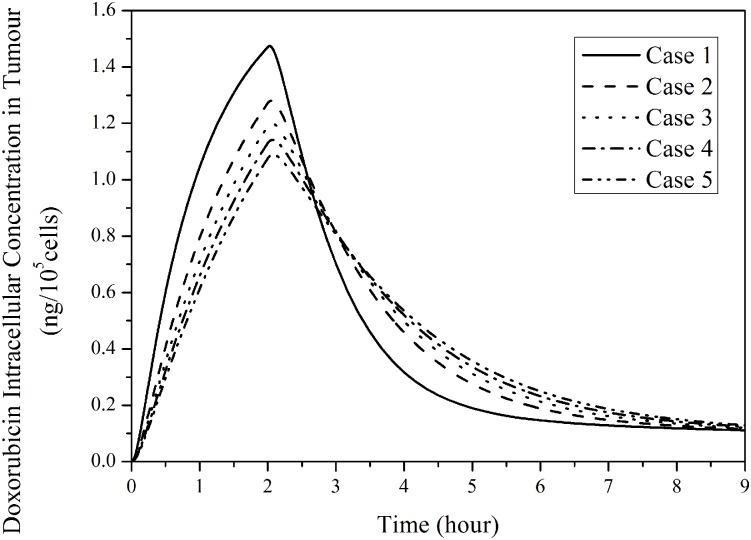
Temporal profiles of predicated intracellular concentration in tumours with different sizes. (2-hour infusion, total dose = 50 mg/m^2^).

Fig 11 presents the intracellular doxorubicin concentration in the five tumours for 2-hour continuous infusion. It follows the trend of free doxorubicin extracellular concentration in tumour as shown in Fig 8(a). The computational model predicts that small tumours with a denser microvasculature have a more rapid changing rate and higher peak. However, intracellular concentration in a large tumour sustains at a slightly higher level after drug infusion ends.

### Doxorubicin cytotoxic effect

**Fig 12:**
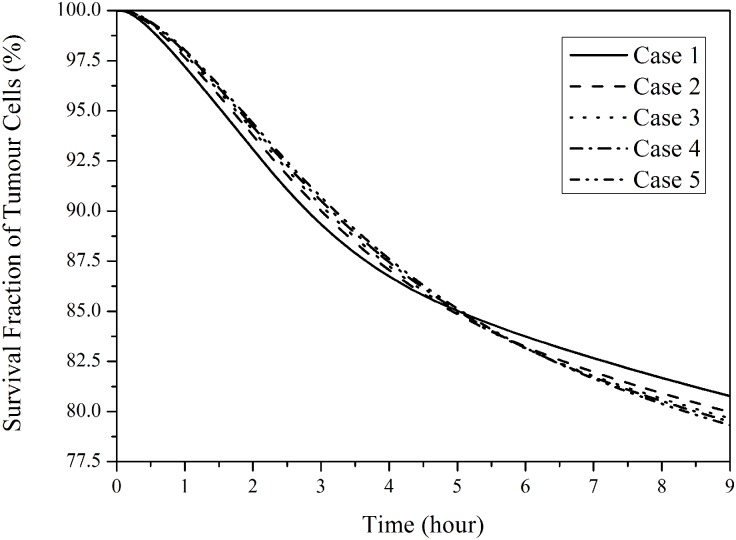
Survival cell fraction of tumour as a function of time in five tumours with different sizes. (2-hour infusion, total dose = 50 mg/m^2^).

Fig 12 presents the fraction of survival tumour cells by applying a suitable pharmacodynamics model [14]. In small tumours with a denser vasculature, more tumour cells can be killed in the first few hours after administration begins. However, the cytotoxic effect of drug in small tumours weakens as time proceeds. This is because in small tumours intracellular drug concentration increases rapidly to a higher peak value, but also decreases faster (as shown in Fig 11), leading to ineffective cell killing during late hours of a treatment. Nevertheless, the predicted difference in cytotoxic effectiveness for the five tumours is small, with a difference of up to 3.2% between Case 1 (smallest tumour) and Case 5 (largest tumour) in the simulation duration.

## Discussion

Interstitial fluid pressure (IFP) in tumour and its surrounding normal tissue plays an important role in determining not only the convective drug exchange between microvessels and the interstitial space driven by transvascular pressure gradient, but also drug convection in extracellular space. Computational simulation results show that tumour IFP is uniform except at the boundary in all models regardless of their sizes. This is because IFP equilibrates with the effective vascular pressure, which is governed by *p*_*v*_ − *σ*_*p*_(*π*_*v*_ − *π*_*i*_) in the tumour interior. This rather uniform IFP dictates that pressure-induced interstitial drug migration is weak, and convective transport in the interstitium only occurs within a thin layer at the tumour periphery, where a steep pressure gradient exists.

The ratio of interstitial to vascular resistances to fluid flow can be measured by a dimensionless parameter, *α*, which is defined as

α=RKvSVK (2)

where *R* is the equivalent radius of the tumour, *K*_*v*_ is the hydraulic conductivity of the microvascular wall, and *K* is the hydraulic conductivity of the interstitium, which is given by *K* = *κ*/*μ* (see S2 Table for definition of symbols and values used). Higher *α* corresponds to higher interstitial flow resistance than that of vascular wall. Using the definition given in Eq (2), values for *α* are 9.42, 18.88, 23.37, 26.89 and 30.45 for Case 1 ~ 5, respective, showing increasing *α* with tumour size. It has been reported that as *α* increases, the IFP profile becomes steeper at the tumour periphery giving rise to increased fluid loss from the tumour [12]. It has also been found that when *α* is greater than 6.32, which is exceeded in all the cases examined here, the above effect becomes obvious and the results become less sensitive to *α* [7]. All these findings are consistent with the results shown here that spatial mean convective drug transport is weaker in large tumours, mainly due to reduced transvascular exchange in the tumour interior.

On the other hand, our numerical results demonstrate that the main transport mechanism for transvascular exchange of drug is diffusion, which strongly depends on (1) the microvasular density and (2) the concentration gradient across the vessel wall. The former is responsible for the higher tranvascular exchange rate observed in small tumours [7, 21, 22]. However, when drug infusion ceases, the direction of diffusion is reversed (from outward to inward), resulting in a fall in interstitial drug concentration. Since small tumours have a dense microvasculature, they lose drug more rapidly than large tumours, which may cancel out the gain in increased extravasation of drug during drug infusion. Although Sefidgar *et al* [13] reported similar finding that the peak extracellular drug concentration decreases with the increase of tumour size, their study did not include the effect of tumour size-dependent microvascular density on drug transport.

It has also been reported that tumour IFP approximately equals to the surrounding pressure for very small tumours with an equivalent radius of less than 0.2 mm [12]. Convection in such small tumours is expected to be strong and hence drug transport mechanisms will be different from that discussed here. It should be noted that the mathematical model adopted in the present study would not be valid for very small tumours whose sizes are of the same order of magnitude as the inter-capillary distance [4, 23]. A tumour cord model [17] or more complex models with explicit representation of capillary vessels would be required in those cases.

The mathematical model employed here has a number of limitations. Firstly, the tumour microvascular network is treated as a distributed source term in the governing equations instead of being modelled explicitly. Hence, the influence of geometric features of tumour vasculature on drug delivery is not included. Secondly, values for *S/V* (blood vessel surface area to tumour volume ratio) and its tumour size dependence are derived from data reported on breast tumour, due to the lack of such information on prostate tumour. Thirdly, uniform transport properties and uniform tumour cell density [7–10, 15–18, 24–34] are assumed in all the tumour models, without accounting for intra- or inter-tumour heterogeneity. Finally, tumour angiogenesis is a dynamic and complex process involving several cellular and subcellular events in *vivo* [35], and the administered doxorubicin can inhibit angiogenesis rather than exerting cytotoxic effect on tumour cells in doxorubicin-insensitive tumour models [36–38]. Changes in tumour vasculature are expected to directly affect drug concentration in interstitial fluid and cell killing. However, it has been reported that dynamic variation of vasculature in prostate tumour is quite slow, and the role of angiogenesis in prostate cancer remains controversial [39]. Because of the aforementioned limitations and the lack of experimental validation, the model predictions described in this study should be regarded as qualitative rather than quantitative. It should also be noted that although the 3-D tumour model geometries are extracted from patient-specific MR images of prostate tumours, other model parameters are neither patient- nor tumour-specific.

## Conclusions

The transport of doxorubicin in five tumours with different sizes and microvasculature densities has been studied under direct continuous infusion. Computational simulation results demonstrate nonlinear relationships between spatial-mean interstitial fluid pressure and tumour volume, as well as between transvascular flux per tumour volume and tumour volume. During drug infusion, transvascular drug exchange depends mainly on diffusion, driven by the concentration gradient of unbound drug between blood and interstitial fluid. As a consequence, transvascular transport is more efficient in small tumours, owing to the low spatial-mean interstitial fluid pressure and dense microvasculature. However, anticancer effectiveness appears to be compromised in small tumours as a result of enhanced reverse diffusion of drug to the blood circulation after the cessation of drug infusion. Future computational studies should aim to incorporate tumour-specific properties in order to understand the quantitative effect of tumour size on treatment outcomes for different tumour types.

## Supporting information

S1 Table Mathematical model. (DOCX)

S2 Table Parameters for tumour and normal tissues. (DOCX)

S3 Table Parameters for doxorubicin. (DOCX)
