# Supplementary material for: The effect of tumour size on drug transport and uptake in 3-D tumour models reconstructed from magnetic resonance images
Source: PLoS One. 2017 Feb 17;12(2):e0172276. doi: 10.1371/journal.pone.0172276 (PMC5315397; doi:10.1371/journal.pone.0172276)
Supplement: S1 Table — (DOCX) [file pone.0172276.s001.docx]

S1 Table. Mathematical model

| Interstitial fluid flow |
| --- |
| $\nabla\cdot v=F_{v}-F_{ly}$; $F_{v}=K_{v}\frac{S}{V}\left[p_{v}-p_{i}-\sigma_{T}\left(\pi_{v}-\pi_{i}\right)\right]$; $F_{ly}=K_{ly}\frac{S_{ly}}{V}\left(p_{i}-p_{ly}\right)$ |
| $\frac{\partial\left(\rho\mathbf{v}\right)}{\partial t}+\nabla\cdot\left(\rho\mathbf{vv}\right)=-\nabla p+\nabla\cdot\tau+W\mu\mathbf{v}$; $W=\frac{1}{\kappa}$ |
| Free doxorubicin concentration in the extracellular space (*C_fe_*) * |
| $\frac{\partial C_{fe}}{\partial t}+\nabla\cdot\left(C_{fe}\mathbf{v}\right)=D_{fe}\nabla^{2}C_{fe}+F_{v}\left(1-\sigma_{d}\right)C_{fp}+P_{fe}\frac{S}{V}\left(C_{fp}-C_{fe}\right)\frac{\text{Pe}_{\text{f}}}{e^{\text{Pe}_{\text{f}}}-1}-F_{ly}C_{fe}+k_{d}C_{be}-k_{a}C_{fe}+D_{c}V_{\max}\left(\frac{C_{i}}{C_{i}+k_{i}}-\frac{C_{fe}}{C_{fe}+k_{e}\phi}\right)$ |
| Bound doxorubicin concentration in the extracellular space (*C_be_*) |
| $\frac{\partial C_{be}}{\partial t}+\nabla\cdot\left(C_{be}\mathbf{v}\right)=D_{be}\nabla^{2}C_{be}+F_{v}\left(1-\sigma_{d}\right)C_{bp}+P_{be}\frac{S}{V}\left(C_{bp}-C_{be}\right)\frac{\text{Pe}_{\text{b}}}{e^{\text{Pe}_{\text{b}}}-1}+k_{a}C_{fe}-k_{d}C_{be}$;$\text{Pe}_{\text{b}}=\frac{F_{v}\left(1-\sigma_{d}\right)}{P_{be}S/V}$ |
| Doxorubicin intracellular concentration (*C_i_*) |
| $\frac{\partial C_{i}}{\partial t}=V_{\max}\left(\frac{C_{fe}}{C_{fe}+k_{e}\phi}-\frac{C_{i}}{C_{i}+k_{i}}\right)$ |
| Pharmacodynamics model |
| $\frac{dD_{c}}{dt}=-\frac{f_{\max}C_{i}}{EC_{50}+C_{i}}D_{c}+k_{p}D_{c}-k_{g}D_{c}^{2}$ |

*Pe_f_ is defined in the same way as Pe_b_
