# Supplementary material for: The effect of tumour size on drug transport and uptake in 3-D tumour models reconstructed from magnetic resonance images
Source: PLoS One. 2017 Feb 17;12(2):e0172276. doi: 10.1371/journal.pone.0172276 (PMC5315397; doi:10.1371/journal.pone.0172276)
Supplement: S2 Table — (DOCX) [file pone.0172276.s002.docx]

S2 Table. Parameters for tumour and normal tissues.

| Parameter | Definition | Unit | Tumour Tissue | Normal Tissue | Reference |
| --- | --- | --- | --- | --- | --- |
| *S/V* | Surface area of blood vessels per unit tissue volume | m^-1^ | 20000 | 7000 | [[7-10](#_ENREF_7)] |
| *K_v_* | Hydraulic conductivity of the micro-vascular wall | m/Pa·s | 2.10×10^-11^ | 2.70×10^-12^ | [[7-10](#_ENREF_7)] |
| *ρ* | Density of interstitial fluid | kg/m^3^ | 1000 | 1000 | [[10](#_ENREF_10)] |
| *µ* | Dynamic viscosity of interstitial fluid | kg/m·s | 0.00078 | 0.00078 | [[10](#_ENREF_10)] |
| *κ* | Darcy’s permeability of the interstitial space | m^2^ | 2.29×10^-17^ | 4.52×10^-18^ | [[7-10](#_ENREF_7)] |
| *p_v_* | Vascular fluid pressure | Pa | 2080 | 2080 | [[7-10](#_ENREF_7)] |
| *π_v_* | Osmotic pressure of the plasma | Pa | 2666 | 2666 | [[7-10](#_ENREF_7)] |
| *π_i_* | Osmotic pressure of interstitial fluid | Pa | 2000 | 1333 | [[7-10](#_ENREF_7)] |
| *σ_T_* | Average osmotic reflection coefficient for plasma proteins |  | 0.82 | 0.91 | [[7-10](#_ENREF_7)] |
| *K_ly_S_ly_/V* | Hydraulic conductivity of the lymphatic wall times surface area of lymphatic vessels per unit volume of tumour tissue | (Pa·s)^-1^ | 0 | 4.17×10^-7^ | [[10](#_ENREF_10)] |
| *p_ly_* | Intra-lymphatic pressure | Pa | 0 | 0 | [[10](#_ENREF_10)] |
| *D_c_* | Cell density | 10^5^cell/m^3^ | 1×10^10^ | - | [[17](#_ENREF_17)] |
| *V_T_* | Total tumour volume | m^3^ | 5×10^-5^ | 3×10^-4^ | [[18](#_ENREF_18)] |
| *V_B_* | Total blood volume in body | m^3^ | 5×10^-2^ | 5×10^-2^ | [[18](#_ENREF_18)] |
