# Supplementary material for: The effect of tumour size on drug transport and uptake in 3-D tumour models reconstructed from magnetic resonance images
Source: PLoS One. 2017 Feb 17;12(2):e0172276. doi: 10.1371/journal.pone.0172276 (PMC5315397; doi:10.1371/journal.pone.0172276)
Supplement: S3 Table — (DOCX) [file pone.0172276.s003.docx]

S3 Table. Parameters for doxorubicin

| Parameter | Definition | Unit | Free Doxorubicin | Bound Doxorubicin | Reference |
| --- | --- | --- | --- | --- | --- |
| *P_tumour_* | Permeability of vasculature wall in tumour | m/s | 3.00×10^-6^ | 7.80×10^-9^ | [[10](#_ENREF_10), [24](#_ENREF_28)] |
| *P_normal_* | Permeability of vasculature wall in normal tissue | m/s | 3.75×10^-7^ | 2.50×10^-9^ | [[10](#_ENREF_10), [24](#_ENREF_28)] |
| *D_tumour_* | Diffusion coefficient in interstitial fluid of tumour | m^2^/s | 3.40×10^-10^ | 8.89×10^-12^ | [[10](#_ENREF_10), [25-29](#_ENREF_29)] |
| *D_normal_* | Diffusion coefficient in interstitial fluid of normal tissue | m^2^/s | 1.58×10^-10^ | 4.17×10^-12^ | [[10](#_ENREF_10), [25-29](#_ENREF_29)] |
| *σ_d_* | Osmotic reflection coefficient |  | 0.15 | 0.82 | [[10](#_ENREF_10), [30](#_ENREF_34)] |
| *k_a_* | Doxorubicin-protein binding rate | s^-1^ | 0.833 | - | [[17](#_ENREF_17)] |
| *k_d_* | Doxorubicin-protein dissociation rate | s^-1^ | - | 0.278 | [[17](#_ENREF_17)] |
| *φ* | Tumour fraction extracellular space |  | 0.4 |  | [[17](#_ENREF_17)] |
| *V_max_* | Rate of trans-membrane transport | kg/10^5^cells s | 4.67×10^-15^ | - | [[17](#_ENREF_17), [31](#_ENREF_35)] |
| *k_e_* | Michaelis constant for transmembrane transport | kg/m^3^ | 2.19×10^-4^ | - | [[17](#_ENREF_17), [31](#_ENREF_35)] |
| *k_i_* | Michaelis constant for transmembrane transport | kg/10^5^cells | 1.37×10^-12^ | - | [[17](#_ENREF_17), [31](#_ENREF_35)] |
| *f_max_* | Cell-kill rate constant | s^-1^ | 1.67×10^-5^ | - | [[32](#_ENREF_36)] |
| *EC_50_* | Drug concentration producing 50% of *f_max_* | kg/10^5^cells | 5×10^-13^ | - | [[32](#_ENREF_36)] |
| *A_1_* | parameter for pharmacokinetic model | m^-3^ | 74.6 | 74.6 | [[17](#_ENREF_17), [33](#_ENREF_37)] |
| *A_2_* | parameter for pharmacokinetic model | m^-3^ | 2.49 | 2.49 | [[17](#_ENREF_17), [33](#_ENREF_37)] |
| *A_3_* | parameter for pharmacokinetic model | m^-3^ | 0.552 | 0.552 | [[17](#_ENREF_17), [33](#_ENREF_37)] |
| *α_1_* | compartment clearance rate | s^-1^ | 2.43×10^-3^ | 2.43×10^-3^ | [[17](#_ENREF_17), [33](#_ENREF_37)] |
| *α_2_* | compartment clearance rate | s^-1^ | 2.83×10^-4^ | 2.83×10^-4^ | [[17](#_ENREF_17), [33](#_ENREF_37)] |
| *α_3_* | compartment clearance rate model | s^-1^ | 1.18×10^-5^ | 1.18×10^-5^ | [[17](#_ENREF_17), [33](#_ENREF_37)] |
| *k_p_* | Cell proliferation rate | s^-1^ | 3.0×10^-6^ | - | [[34](#_ENREF_38)] |
| *k_g_* | Cell physiologic degradation rate | s^-1^ | 3.0×10^-16^ | - | [34] |
